# Volunteering Predicts Mixed Outcomes on Subjective Wellbeing

**DOI:** 10.3390/bs16091610

**Published:** 2026-09-09

**Authors:** Helen Keyes, Sarah Gradidge, Caoimhe Ryan, Eimear Mary Lee

**Affiliations:** 1 School of Psychology, Sport and Sensory Sciences, Anglia Ruskin University, Cambridge CB1 1PT, UK; sarah.gradidge@aru.ac.uk (S.G.); eimear.lee@aru.ac.uk (E.M.L.); 2 Department of Psychology, Glasgow Caledonian University, Glasgow G4 0BA, UK; caoimhe.ryan@gcu.ac.uk

**Keywords:** volunteering, subjective wellbeing, loneliness

## Abstract

This study explored whether engaging in volunteering predicted subjective wellbeing and loneliness, above and beyond known sociodemographic predictors. Secondary data from 7146 adults from the Taking Part 2019–2020 survey (UK household survey of participation in culture and sport) were analysed. Hierarchical regressions were used to explore whether engaging in volunteering (yes/no) predicts subjective wellbeing variables (a sense that life is worthwhile, happiness, life satisfaction and anxiety) and loneliness. Gender, age group, Index of Multiple Deprivation (IMD), health and employment status were included as covariates. Volunteering significantly and positively predicted a sense that life is worthwhile, although with small variance explained, and was also unexpectedly associated with greater anxiety. These relationships were observed above and beyond the variance explained by the known sociodemographic predictors. Our study provides some support for the idea that volunteering may be linked to greater wellbeing, in terms of life being worthwhile, but offers a note of caution about the potential negative relationship with anxiety. Based on our findings, we suggest that volunteering is not necessarily linked to greater hedonic wellbeing but may instead be linked to greater eudaimonic wellbeing, that is, a sense that life is worthwhile.

## 1. Introduction

Volunteering has long been found to be associated with a variety of positive wellbeing indicators including higher life satisfaction, happiness and sense of meaning alongside improved physical and mental health (Nichol et al., 2024; J. W. Yeung et al., 2018). Defined as engagement in unpaid activities through an individual’s free choice (Salamon & Sokolowski, 2016), volunteering can occur across a range of third-sector areas including charities, community organisations and health and social care settings. Volunteering is regarded as a net benefit to society, meeting unmet needs of individuals and communities, bolstering, complementing, or filling gaps left by formalised and governmental social provision. Volunteers’ economic contribution to society is substantial, with the UN (2015) estimating that volunteer activity accounts for approximately 2.5% of global GDP.

Moreover, there may be positive wellbeing outcomes for beneficiaries of volunteering. A review of twenty-two studies found that volunteering activities, particularly befriending and peer support, were associated with increased sense of social participation, self-esteem, and agency, and reduced loneliness, amongst beneficiaries (Grönlund & Falk, 2019). Despite this previous work, some questions remain as to the benefits of volunteering and whether volunteering is always associated with positive wellbeing.

### 1.1. Benefits to Volunteers

Research shows that, rather than the associated benefits of volunteering being limited to those whom the work is assisting, numerous benefits seem to also be apparent for the mental and physical health and wellbeing of volunteers themselves. For instance, descriptive data from a recent National Council for Voluntary Organisations (NCVO) survey offers a useful insight into the opinions of volunteers themselves regarding the personal benefits of volunteering (National Council for Voluntary Organisations, 2020). Over 90% of volunteers in the UK enjoy their volunteer activities and feel they are making a difference, with 77% reporting that they feel volunteering improves their mental health and wellbeing (National Council for Voluntary Organisations, 2020).

A recent umbrella review assessing the effects of volunteering across a range of contexts found small but consistent positive effects of volunteering on a variety of outcomes across the domains of physical, psychological and social wellbeing (Nichol et al., 2024). Studies were primarily US-based with a general focus on older volunteers. More specifically, subjective wellbeing, understood in a broad sense as individuals’ emotional and affective experiences and evaluative judgements such as life-satisfaction (Diener et al., 1999), is a central concern within volunteerism literature and the primary focus of the current paper. While there is some evidence that people who are more satisfied with life are more likely to take on volunteering roles (Lawton et al., 2021), longitudinal data from the UK (Binder, 2015; Tabassum et al., 2016) and the US (Borgonovi, 2008; Choi & Kim, 2011; Piliavin & Siegl, 2007) indicates that moderate and sustained volunteering is associated with improved subjective wellbeing over time. When those with lower baseline life satisfaction do volunteer, they see the highest gains in wellbeing improvement (Binder, 2015).

Associated wellbeing benefits of volunteering are considered robust enough in British policy to warrant estimates of the monetary value of these wellbeing gains by governmental and non-governmental agencies. Using the WELLBY (Wellbeing-Adjusted Life Year) approach, it has been estimated that the increase in wellbeing associated with volunteering is equivalent to an income increase of approximately £673 to £911 per year for every volunteer in England (Department for Culture, Media and Sport, 2025; Lawton et al., 2021) or up to £1000 in Scotland (Volunteer Scotland, 2023).

The limited experimental research in this domain has found that taking part in a specific volunteering programme improved life satisfaction but not psychological wellbeing or empowerment amongst refugee women in Jordan (Panter-Brick et al., 2024). Elsewhere, there is evidence that structured volunteering can improve life satisfaction and other positive outcomes following traumatic brain injury (Payne et al., 2020). Experimental results are not uniform, however; one experimental study found null results with respect to volunteering in a sample of younger adults in North America (Whillans et al., 2016), whereby no difference in subjective wellbeing indicators was found when a sample of volunteers was compared to non-volunteers.

### 1.2. Affect

In seeking to understand precise ways in which volunteering is associated with improved subjective wellbeing, we can separate relationships between volunteering and affective vs. evaluative domains of wellbeing. There is consistent evidence to indicate that volunteering is associated with a reduction in negative affective outcomes including depressive symptoms and psychological distress (Anderson et al., 2014; Nichol et al., 2024; Piliavin & Siegl, 2007; Tabassum et al., 2016; J. W. Yeung et al., 2018). By contrast, there is little evidence regarding relationships between volunteering and happiness (or similar positive affect variables), except where happiness is operationalised as ‘life satisfaction’ (c.f., Binder, 2015; Borgonovi, 2008; Lawton et al., 2021; Lee, 2018). For instance, when explored in a large international sample of older adults, volunteering was linked to improved life satisfaction but not happiness (Gil-Lacruz et al., 2019). One way in which volunteering may theoretically reduce poor affective outcomes is by buffering the effects of stress. For instance, while Han et al. (2020) found no direct link between volunteering and affect, they nevertheless found that the negative emotional impact of daily stressors on affect was less on days when participants volunteered, consistent with the idea that volunteering functions as a psychological and social resource that buffers against stress. Recently, research with student volunteers found that satisfaction with volunteering was associated with perceived emotional benefits of volunteering, rather than more extrinsic benefits such as professional development, suggesting that emotional benefits of volunteering may be recognised by volunteers themselves and function as conscious motivation (Montagud Mayor & Martin Estalayo, 2025).

Alongside these affective processes, there are strong arguments to be made that the ways in which volunteers experience and interpret the meaningfulness of their volunteer work may be important. Motivations for volunteering are varied and can encompass both other-oriented (consistent with altruistic or humanitarian intentions) and self-oriented (where there is a greater emphasis on reciprocity or the potential transactional nature of the work, such as career development) motivations (J. W. Yeung et al., 2018). Both other- and self-oriented volunteering are associated with wellbeing, but there is evidence that other-oriented volunteering is associated with stronger positive wellbeing for the volunteer as compared to self-oriented volunteering, perhaps reflecting the importance of the meaning volunteers ascribe to their work in promoting wellbeing (J. W. Yeung et al., 2018). In one study of hospital volunteers, the more time volunteers spent on volunteering activities, the greater the sense of purpose and wellbeing expressed by participants (Thoits, 2012). The authors posited that the salience of the volunteering role to an individual was important to understand, suggesting that greater personal perceptions of overall purpose and meaning were key to understanding better health outcomes.

Greenfield and Marks (2004) found that in older adults (aged 65–74), there was a positive relationship between formal volunteering and wellbeing, which the authors posited was likely due to the role identity associated with volunteering, with a volunteering role offering a protective route to enhance sense of purpose in life. Interestingly, in this study, the authors posit that the relationship between volunteering and wellbeing is complex and likely multidimensional, in that, irrespective of volunteering, increased negative affect and decreased positive affect were evident in older age. Also, amongst older adults, Chung et al. (2020) found that higher life satisfaction was associated with volunteering roles that involved greater social, intellectual and emotional engagement, with less demanding roles linked to lower life satisfaction and more depressive symptoms. Beyond providing mental stimulation, a further explanation of this finding could be that more engaging forms of volunteering could offer greater opportunities for experiences of purpose or meaning. Volunteering may offer opportunities to rebuild a sense of purpose where this has been lost. For instance, based on qualitative research, Pérez-Corrales et al. (2019) argue that volunteering may offer people with mental disorders the opportunity to acquire responsibility and find meaning in work.

### 1.3. Loneliness

In addition to affective and evaluative processes, there is reason to believe that processes relating to social connectedness may also be important in understanding the relationship between volunteering and subjective wellbeing. In volunteering one’s time, there are quite often opportunities for development of social ties with like-minded individuals and the potential to facilitate purpose and meaning through shared senses of belonging. Indeed, there is evidence that the number of social groups with which people identify predicts their level of wellbeing, and volunteering provides opportunity for increased identification (Ripley-McNeil & Cramer, 2020). Qualitative research with volunteers in England found that experiencing shared identity with fellow volunteers was felt to provide a sense of belonging amongst volunteers, which in turn was experienced as important for volunteers’ sense of wellbeing (Gray & Stevenson, 2020). On this basis, volunteering might provide particular benefits to those experiencing social isolation and loneliness. In their longitudinal work, Piliavin and Siegl (2007) found that wellbeing benefits of volunteering were moderated by social integration, whereby volunteers who were less socially integrated prior to volunteering benefited the most.

A number of recent studies have found that volunteering can reduce loneliness in older adults (Cho & Xiang, 2023; Lee, 2022; Richardson et al., 2025; D. Y. L. Yeung et al., 2025). In particular, D. Y. L. Yeung et al. (2025) report that these benefits can be maintained through ongoing engagement with volunteering. Meanwhile, Cho and Xiang (2023) examined the relationship between volunteering and loneliness over a 12-year follow-up period, offering a notable longitudinal perspective. Shorter-term longitudinal research found that starting a volunteering role was associated with a small but significant decrease in feelings of loneliness, though it was not associated with broader perceptions of social connectedness. However, there is limited research on whether this reduction in loneliness would be observed across a wider age range, considering that younger age groups may have more frequent opportunities for interaction in their daily lives.

### 1.4. Psychological Cost of Volunteering

It is important to note that volunteering is not a universally positive experience and comes with psychological costs as well as benefits. A large minority of UK volunteers report feeling pressured or unappreciated in their volunteer roles, with negative experiences more common amongst younger volunteers and volunteers with disabilities (National Council for Voluntary Organisations, 2020). Moreover, by their very nature, some volunteer roles expose individuals to emotionally and psychologically challenging circumstances. For example, research has found that crisis line volunteering can lead to short-term increases in psychological distress and functional impairment (Kitchingman et al., 2018). These effects appear to differ depending on personal factors such as mental health history, indicating that psychological costs depend on individual and contextual factors. Meanwhile, Gellweiler et al. (2018) found that volunteers experienced loss and sadness at the conclusion of their temporary sport volunteer roles, offering an important reminder of the often short-term or intermittent nature of some voluntary roles which may confer their own hidden costs.

### 1.5. Present Study, Aims and Hypotheses

There is substantial evidence that volunteering is associated with psychological and social benefits for volunteers. However, most of the evidence for this relationship derives from studies focused on specific forms of volunteering, specific types or levels of engagement, or particular populations, most notably older adults. Substantive syntheses exist but by their nature are necessarily characterised by substantial heterogeneity in the operationalisation of predictors and outcomes, as well as variation in study populations and designs (e.g., Anderson et al., 2014; Nichol et al., 2024). Whilst UK population-level evidence has linked volunteering to positive wellbeing outcomes, studies focus specifically on frequency of volunteering and operationalise wellbeing in specific and limited ways, including life satisfaction only (Binder, 2015) and mental wellbeing as measured by the GHQ12 (Tabassum et al., 2016). Taken together, it remains unclear whether reported associations reflect robust, population-level relationships or are specific to certain populations, contexts, or forms of volunteering. It remains to be seen whether associations between volunteering and subjective wellbeing outcomes persist under simple, parsimonious empirical conditions.

The current study controls for sociodemographic variables that are already known to be linked to subjective wellbeing and loneliness outcomes (Keyes et al., 2023; Keyes et al., 2024): gender, age, level of deprivation, health and employment status. For example, poor health (Jessen et al., 2017; Steptoe et al., 2015), deprivation (Bellis et al., 2012; De Koning et al., 2016) and unemployment (Bonanomi & Rosina, 2022; Morrish & Medina-Lara, 2021) have been associated with poorer wellbeing and increased loneliness. Gender and age have also both been shown to be linked to subjective wellbeing (Batz & Tay, 2018; Inoue et al., 2020; López Ulloa et al., 2013).

In light of this, these variables could plausibly impact volunteering engagement, either through motivation (age, gender) or available time and resources (deprivation, employment, health). We include these variables in our analyses to (1) control for them as potential candidate confounders, (2) control for the variance explained by these known sociodemographic predictors of wellbeing and loneliness, and (3) assess the relationships between volunteering and subjective wellbeing and loneliness *above and beyond* these known sociodemographic predictors. In this way, we can assess to what extent volunteering predicts subjective wellbeing and loneliness as compared to the relative effect sizes of the sociodemographic predictors.

In the present study, we use large-scale, representative UK data (Taking Part survey, 2019–2020) and widely adopted national indicators of wellbeing (ONS4; sense of meaning in life, life satisfaction, happiness, anxiety) to assess whether any recent engagement with volunteering, regardless of domain, is associated with higher levels of key subjective wellbeing outcomes, as well as loneliness. By using a parsimonious measure of volunteering, a conceptually robust measure of wellbeing, and an up-to-date, representative dataset, we aim to make an important contribution to understanding the relationships between volunteering and subjective wellbeing and loneliness at a population level.

**H1.**  *Engagement in volunteering will predict greater subjective wellbeing, whereby there is greater sense that life is worthwhile (a), greater life satisfaction (b), greater happiness (c), and lower anxiety (d), over and above other sociodemographic factors.*

**H2.**  *Engagement in volunteering will predict lower loneliness over and above other sociodemographic factors.*

## 2. Method

### 2.1. Participants

The current study utilised a secondary dataset from the Taking Part Survey (Year 13: April 2019–March 2020), which is a face-to-face household survey conducted by the Department for Digital, Culture, Media and Sport. The sample is a random, representative sample, with all participants being aged 16 or over and living in England. The data is available at the UK Data Service: http://doi.org/10.5255/UKDA-SN-8745-1. For the current analysis, out of the 7502 participants in the original dataset, 7146 participants were retained as the final sample for analyses: One participant (0.013%) was removed due to having a gender identity other than male or female, as we could neither include this participant in dummy coding as ‘male’ or ‘female’, nor introduce a third dummy-coded gender category as there was only one participant identifying as neither male nor female, thereby meaning there was insufficient data statistically and representatively for this gender identity group. An additional 355 (4.73%) participants were removed due to missing data on predictor or outcome variables. Such missing data were found to be missing completely at random, *p* > 0.05, and the demographic characteristics of the excluded sample are highly similar to the final sample. Sociodemographic characteristics of the final sample are represented in Table 1.

### 2.2. Materials

#### 2.2.1. Demographics

Participants reported demographic details of age (measured in groups of aged 16–19, 20–24, 25–34, 35–44, 45–54, 55–64, 65–74, 75–84, and 85+), gender (measured as male or female), employment status (working or not working), level of general health (measured through answer on scale from 1 ‘*very good*’ to 5 ‘*very bad*’ to the question *‘How is your health in general?’*), and level of deprivation (assessed by participant’s home postcode, which is part of the Index of Multiple Deprivation [IMD], measured from 1 ‘*most deprived*’ to 10 ‘*least deprived*’).

#### 2.2.2. Engagement in Volunteering

Engagement in volunteering was measured categorically, with participants who stated they had engaged in any of 18 different volunteering activities listed within the past 12 months being classified as ‘engaged in volunteering’, and any participants who stated they had not engaged in any of these 18 activities within the past 12 months being classified as ‘not engaged in volunteering’. Specifically, participants were asked the question ‘*During the last 12 months, have you done any of following kinds of voluntary work?*’, with the following 18 volunteering activities: raising or handling money/taking part in sponsored events; leading a group; member of a committee; trustee; organising or helping to run an activity or event; steward at a heritage site/museum or gallery; visiting people; befriending/mentoring people; coaching or tuition; giving advice/information/counselling; secretarial, administrative or clerical work; providing transport or driving; representing, e.g., addressing meetings, leading a delegation; campaigning, e.g., lobbying, canvassing, letter writing; conservation/restoration; officiating, e.g., judging, umpiring or refereeing; other practical help, for example, helping out a school, religious group, with shopping/refreshments; and other (please specify).

#### 2.2.3. Subjective Wellbeing

Subjective wellbeing was measured through four single-item questions on a Likert scale from 0 to 10, assessing anxiety, life satisfaction, a sense that life is worthwhile, and happiness. Anxiety was measured through the question ‘*On a scale where 0 is “not at all anxious” and 10 is “completely anxious,” overall, how anxious did you feel yesterday?*’, life satisfaction through ‘*Overall, how satisfied are you with your life nowadays?” (0 = “not at all satisfied” to 10 = “completely satisfied*’, a sense that life is worthwhile through ‘*To what extent do you feel that the things in your life are worthwhile?” (0 = “not at all worthwhile” and 10 = “completely’*, and happiness through ‘*Taking all things together, how happy would you say you are?” (0 = “extremely unhappy” to 10 = “extremely happy*’. Higher scores represent higher levels of that variable. These items were analysed as single-item measures in line with guidance from the Office for National Statistics (ONS, 2018).

#### 2.2.4. Loneliness

Loneliness was measured with a single-item question asking ‘*How often do you feel lonely?*’ with response options of 1 ‘*often or always*’, 2 ‘*some of the time*’, 3 ‘*occasionally*’, 4 ‘*hardly ever*’, and 5 ‘*never*’. Therefore, a higher score indicates lower loneliness.

### 2.3. Procedure

The current study utilises a secondary dataset (the Taking Part Survey), which was a large, representative household survey conducted through face-to-face interviews and run by the UK Department for Culture, Media and Sport. Further detail on the survey can be found at https://www.gov.uk/guidance/taking-part-survey (accessed on 1 February 2026).

Participants were selected randomly through lists of addresses in England, with those randomly selected receiving an invitation to participate via post. An interviewer would then visit the household and interview a randomly selected member of the household aged 16 or over. Participants were then asked a series of questions asking about demographics and engagement in activities related to culture, leisure, heritage and sport. The full list of questions can be found at https://doc.ukdataservice.ac.uk/doc/8745/mrdoc/pdf/8745_taking_part_year_15_adult_child_youth_questionnaire.pdf (accessed on 1 March 2026). Answers to questions would be input digitally by the interviewer, with interviews lasting approximately 45 min.

## 3. Results

### 3.1. Analyses

We used a hierarchical regression model to test to what extent our identified sociodemographic predictor variables (age, gender, level of general health, level of deprivation and employment status) predict outcome variables of subjective wellbeing (happiness, life satisfaction, anxiety, and a sense that life is worthwhile) and loneliness, and whether volunteering predicted changes in these outcome variables above and beyond the variance explained by the sociodemographic variables. We ran a total of five hierarchical regressions, one for each outcome variable. For each analysis, level of deprivation, age group, level of general health, gender and employment status were entered in Block 1, with engagement in volunteering activities entered in Block 2. There was no multicollinearity between the predictor variables, as assessed through VIFs ≤ 1.43. Categorical predictor variables were dummy coded as follows: engagement with volunteering (reference: no engagement); gender (reference: female); employment status (reference: not working). All analyses were carried out using Jamovi software (version 2.7) (The jamovi project, 2025).

### 3.2. Sense That Life Is Worthwhile

Volunteering significantly predicted a sense that life is worthwhile, above and beyond the variance explained by gender, age group, deprivation, general health and employment. Inclusion of volunteering in the model explained an additional 0.4% of variance in participants’ sense that life is worthwhile compared to the variance explained by the sociodemographic variables alone, *F*_(1,7139)_ = 33.2, *p* < 0.001. Volunteering predicted a larger increase in a sense that life is worthwhile (0.250) than being in employment (0.154; see Table 2). In the final stage of the model, gender, level of deprivation, general health, being in employment, age group and volunteering were all significant predictors of a sense that life is worthwhile, with women, older age groups, those living in less deprived areas, those in better health, employed people and people who engaged in volunteering in the last year reporting a higher sense that life is worthwhile. The final model accounted for 11.3% of the variance in sense that life is worthwhile, *R*^2^ = 0.113, *F*_(6,7139)_ = 151, *p* < 0.001. These findings are in line with H1a.

### 3.3. Life Satisfaction

Contrary to H1b, volunteering did not add significantly more predictive power to the model above and beyond the variance explained by the sociodemographic variables. The final model shows that those in better health, those living in areas with less deprivation, those in employment, and those in older age groups reported higher life satisfaction, with 16.5% of the variance in life satisfaction scores being explained by this model, *R*^2^ = 0.165, *F*_(6,7139)_ = 235, *p* < 0.001.

### 3.4. Happiness

Contrary to H1c, the addition of volunteering to the model did not add significantly more predictive power to the model above and beyond the sociodemographic predictors. The final model shows that women, those in better health, and those in older age groups reported being happier, with 10.6% of the variance in happiness scores being explained by this model, *R*^2^ = 0.106, *F*_(6,7139)_ = 141, *p* < 0.001.

### 3.5. Anxiety

Volunteering significantly predicted anxiety scores above and beyond the variance explained by gender, deprivation, age group, general health or being in employment. Here, the inclusion of volunteering in the model accounted for an additional 0.19% of the variance in anxiety scores above and beyond the variance accounted for in stage one of the model, *F*_(1,7139)_ = 14.5, *p* < 0.001. Volunteering predicted a similar increase in anxiety (0.280) as that associated with being in a younger age group (0.274; see Table 2). In the final stage of the model, gender, age group, poor health, and volunteering were all significant predictors of anxiety scores, with men, those in better health, those in older age groups and those who had not volunteered in the last year reporting less anxiety. Approximately 7.5% of the variation in anxiety was accounted for by the final model, *R*^2^ = 0.075, *F*_(6,7139)_ = 97.2, *p* < 0.001. These findings are contrary to H1d, which posited that volunteering would be linked to lower levels of anxiety.

### 3.6. Loneliness

Lastly, and contrary to H2, the addition of volunteering did not provide any additional predictive power to the model when looking at loneliness, above and beyond the sociodemographic predictors included in the first stage. Here, men, those living in less deprived areas, in employment, in better health, and in older age groups reported experiencing less loneliness, with health being the strongest predictor (see Table 2). The final stage of the model accounted for 7.7% of the variance in loneliness scores, *R*^2^ = 0.077, *F*_(6,7139)_ = 99.2, *p* < 0.001.

## 4. Discussion

This study explored whether and to what extent volunteering, regardless of activity type or intensity of engagement, predicted various components of subjective wellbeing (sense that life is worthwhile, life satisfaction, happiness, anxiety) and loneliness in a large, representative, gender-balanced and age-diverse sample from England. A main finding from this study is that engagement in volunteering significantly predicted a greater sense that life is worthwhile, supporting H1a. This finding is in line with previous work, which has evidenced the meaning-based components of volunteering (e.g., Salt et al., 2017), including how volunteering can be conceptualised and experienced as fulfilling a significant purpose (Scott et al., 2021). Unlike the majority of previous work, which explores specific types of volunteering (e.g., healthcare-related volunteering; Tierney et al., 2021), our finding shows a generalised relationship between engagement in any kind of volunteering within the last year and a sense that life is worthwhile. Thus, it appears that the act of volunteering in and of itself is associated with a sense of meaning, and this relationship is not tied to a specific type of volunteering. It is important to note that the variance in a sense that life is worthwhile associated with volunteering is low, which may limit practical significance, although the variance explained by volunteering is more than 1.5 times greater than the variance explained by being in employment. The current findings are not causal, only correlational. However, if future research supports a causal relationship between volunteering and a sense that life is worthwhile, it would be easier to encourage the uptake of volunteering in the population as a way to enhance the sense that life is worthwhile as compared to other intransient variables that are also known to predict wellbeing (e.g., gender, age, deprivation level, health, employment).

It is perhaps unexpected that volunteering predicted greater variance in sense of life being worthwhile than did employment, indicating that, despite the ostensible similarities between volunteering and employment (e.g., work tasks, interaction and teamwork with others, routine), the differences between employment vs. volunteering may lead to differential amounts of variance explained in the sense of life being worthwhile. The key difference may be volunteering entailing freely chosen work without expectation of reward, unlike employment (Nichol et al., 2024), though variables such as number of hours volunteering/in paid employment per week and volunteering/employment sector will need to be controlled for in future research (and note that number of hours may not have an impact; Son & Wilson, 2012). Compared to employment, volunteering may be less likely to be perceived as involving overwork and may actually aid work–life balance (Ramos et al., 2015), with volunteers likely being more able to focus on engaging in voluntary work, which is personally meaningful rather than money-motivated (Rodell, 2013; Schnell & Hoof, 2012). Relatedly, volunteering is also typically in charitable and not-for-profit sectors, with opportunities often not available in employment to have outsized, positive impacts on social causes (Briggs et al., 2010), which may also theoretically contribute to a greater sense of meaning. We should note here, however, that the variance explained by volunteering vs. employment in the sense that life is worthwhile is not a causal finding, so future research would need to determine if volunteering has a larger causal effect on the sense that life is worthwhile than employment.

Except for a sense that life is worthwhile, volunteering was not linked to greater wellbeing, in contrast to previous psychological theory (e.g., volunteering as a form of ‘social prescribing’, Tierney et al., 2021) and evidence (e.g., Nichol et al., 2024). Instead, we found that engagement in volunteering—while being a significant positive predictor of a sense that life is worthwhile—did not significantly predict life satisfaction, happiness, or loneliness above and beyond the variance explained by sociodemographic factors of age, gender, employment status, health and level of deprivation. Interestingly, we found that engagement in volunteering did significantly predict anxiety but not in the hypothesised direction: instead, engagement in volunteering predicted significantly higher levels of anxiety above and beyond the variance explained by known sociodemographic predictors.

The mixed findings reported in this study highlight the importance of considering which type of wellbeing is being measured. Our finding that engagement in volunteering positively predicts a sense of life being worthwhile suggests that volunteering is linked to greater *eudaimonic* wellbeing (sense of meaning) but not to greater *hedonic* wellbeing (presence of positive feelings, e.g., happiness), supporting typically well-established distinctions between the two (see, e.g., Ryff et al., 2021, for a summary). This finding is in line with research that has found similar differential relationships between volunteering and eudaimonic vs. hedonic wellbeing (e.g., Son & Wilson, 2012). The current findings indicate that volunteering is not linked to improved hedonic wellbeing and may even be linked to worse hedonic wellbeing, i.e., heightened negative affect (anxiety). These findings, although not causal, present a contradiction: volunteering may be linked to a greater sense that life is worthwhile whilst also being linked to greater anxiety. If future research finds these relationships are causal, these findings may therefore indicate both a cost (anxiety) and a benefit (sense of life being worthwhile) of volunteering, perhaps reflecting the effortful nature of volunteering. That is, for something to feel purposeful and meaningful, the task may need to feel effortful to achieve this sense of meaning (e.g., Campbell et al., 2025), which may theoretically be linked to heightened activating emotions such as anxiety (Eysenck et al., 2007). Alternatively, it is possible that volunteering may expose individuals to potentially emotionally distressing and emotionally labour-intensive situations, particularly as many volunteering roles are within care-related fields (Tierney et al., 2021), which could increase anxiety (Høgenhaven, 2025) due to role strain and high emotional labour. If the relationships between volunteering and greater sense that life is worthwhile and greater anxiety are proven to be causal, future research should explore whether and how we can encourage volunteering to contribute to both eudaimonic and hedonic wellbeing.

It is also possible that the relationships between volunteering and sense that life is worthwhile and anxiety are not causal. Instead, there may be reverse causality, i.e., it may be that more anxious people tend to volunteer more, perhaps as a way to help with and manage anxiety through social interactions, providing a sense of purpose, and/or distraction. These significant findings must also be interpreted in terms of whether these variables are trait (stable over time) vs. state (variable over time): whilst the ONS does not explicitly state whether their wellbeing variables are state or trait, the sense that life is worthwhile is likely to be trait (measured through the question ‘to what extent do you feel that the things in your life are worthwhile?’) whilst anxiety is likely to be state (measured through ‘on a scale where 0 is “not at all anxious” and 10 is “completely anxious,” overall, how anxious did you feel yesterday?’). As such, the current finding regarding life being worthwhile may be more stable over time, whereas the anxiety finding may not be stable over time. Thus, future research should not only test causality but also disentangle variables on a day-to-day basis through, for instance, experience sampling studies which could measure differences in state anxiety over long time periods while volunteering vs. when not volunteering.

It is interesting that there was no link between engagement in volunteering and life satisfaction, in contrast to previous literature (e.g., Binder, 2015; Jiang et al., 2019). Life satisfaction is often viewed as conceptually different from, yet correlated with, both hedonic and eudaimonic wellbeing (Schmitz et al., 2025); thus, current findings further support a necessary distinction between sense of purpose vs. life satisfaction. Interestingly, volunteering did not predict life satisfaction, yet being in employment did. This difference in findings between volunteering vs. employment again invites us to consider the difference between the two: As employment involves financial gains and (often) financial security, it is intuitive that employment may protect life satisfaction, whilst being unemployed significantly harms life satisfaction (e.g., Chen & Hou, 2019; Richter et al., 2020). In contrast, not engaging in volunteering does not have similar negative implications (i.e., financial insecurity and associated struggle to meet one’s needs) by definition, as volunteering does not provide income.

Surprisingly, there was no significant relationship between volunteering and loneliness, indicating that often proposed social benefits of volunteering may not be occurring (e.g., Gray & Stevenson, 2020). Being in employment, however, did predict lower loneliness, indicating a social distinction between being in employment vs. volunteering. As we only measured whether people had engaged in at least one volunteering activity in the past 12 months, it is possible that people were not engaged in volunteering frequently enough to gain meaningful social benefits (e.g., Schnell & Hoof, 2012).

Our findings on the role of sociodemographic factors in predicting subjective wellbeing and loneliness are in line with previous work (e.g., Keyes et al., 2023; Keyes et al., 2024). Happiness was predicted by health, gender and age, while life satisfaction was predicted by being in employment, level of deprivation, age, and health. Loneliness and a sense that life is worthwhile were both predicted by health, employment, age group and gender, with loneliness being additionally predicted by deprivation. Anxiety was predicted by health, gender and age. In general, being in better health was by far the strongest predictor of greater wellbeing and decreased loneliness and significantly explained variance in all outcome variables. Being in an older age group also significantly explained variance in all outcome variables, predicting greater wellbeing and less loneliness. These results are in line with previous findings for both health and ageing (Hawkley et al., 2020; Jessen et al., 2017; Steptoe et al., 2015). Gender differences in subjective wellbeing and loneliness were mixed, with women reporting being happier and having a greater sense that life is worthwhile, while men reported being less anxious and less lonely. These findings are in line with the mixed literature on gender differences in subjective wellbeing (e.g., Inoue et al., 2020; Mental Health Foundation, 2021).

### 4.1. Limitations and Directions for Future Research

The current study has some limitations. Firstly, due to the cross-sectional nature of the survey, we cannot establish causation in our findings. It is possible that volunteering and wellbeing have a symbiotic relationship, whereby those who have a greater sense of meaning in life or are more anxious are more likely to engage with volunteering. For instance, it is possible that people who have a greater sense of life being worthwhile are in a better life situation with fewer structural barriers to be able to volunteer (‘volunteerability’, Haski-Leventhal et al., 2018). However, there is some evidence to suggest that volunteering is associated with wellbeing gains following engagement (Binder, 2015). Future research should carefully test for causality through, for example, experiments comparing components of wellbeing both pre- and post-volunteering.

Secondly, our significant findings between volunteering and sense that life is worthwhile and anxiety were of small size, which may limit practical significance. Future research should therefore assess longitudinal real-world effects to more precisely determine real-world significance.

Thirdly, due to reliance on a secondary dataset, variables and the ways in which they were measured were beyond the current authors’ control and should be improved in future research. Specifically, the single-item wellbeing and loneliness measures limit psychometric validity and may explain some null findings due to lack of precision in measurement. For instance, participants may not identify themselves as experiencing loneliness when directly asked, whereas a loneliness scale may be better able to identify subtler variations in loneliness and thus any link to volunteering. Additionally, the current study only measured volunteering within the past 12 months in a binary way (yes vs. no), thus conflating those who have never volunteered with those who have volunteered but not in the past 12 months in the ‘no’ category, and conflating those who have volunteered just once in the past 12 months with those who volunteer frequently in the ‘yes’ category. Future research should therefore (a) measure wellbeing and loneliness with psychometrically validated scales and (b) test not just engagement in volunteering (yes vs. no) but also more detailed variables such as frequency, duration, and type of volunteering.

Finally, our study included gender as a known predictor of subjective wellbeing and loneliness; however, due to only one non-binary participant being present in the sample, we could only explore binary gender (male vs. female) without exploration of volunteering and subjective wellbeing and loneliness in people who identify as non-binary. This represents a significant limitation in generalising findings to non-binary people, and future research must address this limitation by oversampling non-binary participants and other gender minorities to allow statistical comparisons in future beyond binary gender.

### 4.2. Strengths and Implications

The current study extends previous literature by utilising a large, representative national sample to highlight how engagement in *any* type of volunteering, regardless of sector, predicts a greater sense of life being worthwhile. Thus, the current findings support the possible meaningfulness of volunteering, in and of itself, regardless of sector-specific activities that volunteering may entail, across a large, gender-balanced and age-diverse sample representative of the broader English population. Aspects of wellbeing which are not meaning-related, i.e., hedonic wellbeing, instead may be of less relevance to volunteering (positive affect) or may be negatively linked to volunteering (negative affect). As discussed in the limitations section, however, our findings are not causal, and significant findings are of small sizes, so practical implications of our study should be treated with caution and followed up with further experimental research.

Our study does, however, have strong theoretical implications for the literature by exploring relationships between volunteering and subjective wellbeing and loneliness in a large, representative sample, and by assessing these relationships as compared to known sociodemographic predictors. That is, assessing the variance explained by volunteering in subjective wellbeing and loneliness above and beyond gender, age, level of deprivation, being in employment, and health allowed for a unique comparison of relative effect sizes between volunteering and these sociodemographic predictors. Our findings especially on the greater variance explained in a sense that life is worthwhile by volunteering as compared to employment possibly indicate psychological differences in volunteering and employment, potentially arising from differences in meaning-making and the opportunity to help others, and freely chosen activity and sense of autonomy, that may be present in volunteering but not in employment.

### 4.3. Conclusions

The current study extends previous research on volunteering by exploring the variance explained by volunteering in subjective wellbeing and loneliness in a large, gender-balanced, and age-diverse representative sample from England, above and beyond known sociodemographic predictors. The results are not causal but do hold key implications: volunteering may be linked to a greater sense that life is worthwhile but not linked to other aspects of wellbeing, indicating differential relationships between volunteering and eudaimonic vs. hedonic wellbeing. In fact, volunteering may even be linked to poorer hedonic wellbeing, as volunteering was associated with higher levels of anxiety. The results also indicate that volunteering was not linked with loneliness, despite purported social benefits of volunteering. The current findings therefore only partially support existing psychological evidence on positive relationships between volunteering and subjective wellbeing (when measured as sense that life is not worthwhile) but do not support positive relationships between volunteering and other aspects of subjective wellbeing or loneliness. From the current study, we suggest that volunteering may not be linked to hedonic wellbeing but might instead be conceptualised as meaning-making and hence associated with eudaimonic wellbeing, that is, a sense that life is worthwhile. Additionally, volunteering may explain greater variance in a sense that life is worthwhile than does employment, indicating possible psychological differences (e.g., free choice and autonomy, meaning-making and opportunity to help others) in volunteering as compared to employment.

## Figures and Tables

**Table 1:** Sociodemographic characteristics of the sample.

Age	16–19: N = 21 (0.3%) 20–24: N = 172 (2.4%) 25–34: N = 925 (12.9%) 35–44: N = 1229 (17.2%) 45–54: N = 1291 (18.1%) 55–64: N = 1285 (18%) 65–74: N = 1223 (17.1%) 75–84: N = 756 (10.6%) 85+: N = 244 (3.4%)
Gender	Female: N = 3884, 54.4% Male: N = 3262, 45.6%
Employment status	Working: N = 3208, 44.9% Not working: N = 3938, 55.1%
General health	M = 2.11, SD = 0.981
Level of deprivation	M = 5.56, SD = 2.86 Frequencies: 1 (relatively most deprived): N = 725 (10.1%) 2: N = 716 (10%) 3: N = 610 (8.5%) 4: N = 647 (9.1%) 5: N = 771 (10.8%) 6: N = 702 (9.8%) 7: N = 769 (10.8%) 8: N = 775 (10.8%) 9: N = 774 (10.8%) 10 (relatively least deprived): N = 657 (9.2%)
Engagement in volunteering	Engagement in volunteering: N = 2264, 31.7% No engagement in volunteering: N = 4882, 68.3%

**Table 2:** Results of two-step hierarchical multiple regressions predicting life satisfaction, happiness, life being worthwhile, anxiety and loneliness with regression coefficients (B) specified for all predictor variables at each step of the regression.

Predictor Variables	Satisfaction	Happiness	Worthwhile	Anxiety	Loneliness
Step 1					
Intercept	8.580 ***	8.350 ***	8.623 ***	2.759 ***	3.789 ***
Gender	−0.077	−0.144 **	−0.234 ***	−0.391 ***	0.210 ***
IMD Decile	0.017 *	0.010	0.019 **	0.024 *	0.020 ***
Age Group	0.119 ***	0.126 ***	0.093 ***	−0.273 ***	0.052 ***
General Health	−0.751 ***	−0.701 ***	−0.573 ***	0.760 ***	−0.283 ***
In employment	0.096 *	0.039	0.152 ***	−0.064	0.194 ***
*R* ^2^	0.165	0.106	0.109	0.074	0.077
*F*	282 ***	169 ***	174 ***	113.6 ***	118.8 ***
Step 2					
Intercept	8.562 ***	8.340 ***	8.546 ***	2.673 ***	3.805 ***
Gender	−0.074	−0.143 **	−0.223 ***	−0.378 ***	0.207 ***
IMD Decile	0.015 *	0.009	0.014	0.019	0.020 ***
Age Group	0.119 ***	0.140 ***	0.092 ***	−0.274 ***	0.052 ***
General Health	−0.749 ***	−0.699 ***	−0.562 ***	0.772 ***	−0.286 ***
In employment	0.096 *	0.004	0.154 ***	−0.062	0.194 ***
Volunteering	0.060	0.034	0.250 ***	0.280 ***	−0.054
*R* ^2^	0.165	0.106	0.113	0.076	0.077
Δ*R*^2^	0.0002	<0.0001	0.0041	0.0019	<0.0004
Δ*F*	1.93	0.43	33.2 ***	14.5 ***	3.21

Note. * *p* < 0.05; ** *p* < 0.01, *** *p* < 0.001.

## Data Availability

The data are publicly available here: Taking Part 2019/20: statistical release—GOV.UK (https://www.gov.uk/government/statistics/taking-part-201920-statistical-release, accessed on 1 February 2026).
